# flowPloidy: An R package for genome size and ploidy assessment of flow cytometry data

**DOI:** 10.1002/aps3.1164

**Published:** 2018-07-23

**Authors:** Tyler William Smith, Paul Kron, Sara L. Martin

**Affiliations:** ^1^ Agriculture and Agri‐Food Canada Neatby Building 960 Carling Avenue Ottawa Ontario K1A 0C6 Canada; ^2^ Department of Integrative Biology University of Guelph Guelph Ontario N1G 2W1 Canada

**Keywords:** flow cytometry, genome size, polyploidy, R package

## Abstract

**Premise of the Study:**

Despite advantages in terms of reproducibility, histogram analysis based on nonlinear regression is rarely used in genome size assessments in plant biology. This is due in part to the lack of a freely available program to implement the procedure. We have developed such a program, the R package flowPloidy.

**Methods and Results:**

flowPloidy builds on the existing statistical tools provided with the R environment. This base provides tools for importing flow cytometry data, fitting nonlinear regressions, and interactively visualizing data. flowPloidy adds tools for building flow cytometry models, fitting the models to histogram data, and producing visual and tabular summaries of the results.

**Conclusions:**

flowPloidy fills an important gap in the study of plant genome size. This package will enable plant scientists to apply a more powerful statistical technique for assessing genome size. flowPloidy improves on existing software options by providing a no‐cost workflow streamlined for genome size and ploidy determination.

Flow cytometry (FCM) has become an indispensable tool for plant scientists studying genome size and polyploidy (Suda et al., 2007a). The principles of the system are straightforward: DNA present in plant tissue samples is rendered fluorescent through the application of selective dyes, after which the amount of DNA in each nucleus can be determined from the amount of light emitted after the tissue is excited with a laser. This process is largely automated via a flow cytometer, allowing an individual researcher to process dozens of samples a day at low cost.

FCM offers many advantages over chromosome squashes, including: high throughput; requires only easily collected leaf tissue, rather than actively dividing root tips or anthers; can be used with silica‐dried tissue; and requires minimal training. As a consequence of its low cost and high throughput, FCM has enabled large‐scale surveys of ploidy at the landscape, population, individual, and tissue levels, with applications in taxonomy, evolution, population biology, ecology, and plant breeding (Kron et al., 2007).

We have developed a new R package, flowPloidy, to fill a gap in the toolchain for the analysis of FCM data by researchers investigating genome size and polyploidy. flowPloidy provides nonlinear‐regression‐based analysis of FCM histograms. Previously, this technique has only been available in expensive proprietary software, contributing to its low adoption in the botanical community. This is unfortunate, as the regression‐based approach minimizes the need for subjective manual gating, making it more robust and repeatable than alternative methods.

In common practice, FCM histogram analysis requires the user to visually isolate histogram peaks from which to extract the parameters used to calculate genome size and associated statistics, including cell counts and the coefficient of variation (CV). This process is inherently subjective; the CV can be lowered by narrowing the gate, thus negating its value in quality control. This process also makes it impossible to distinguish between actual nuclei and background debris, leading to inflated cell counts.

Using nonlinear regression to fit FCM histograms greatly reduces these problems. In this process, we use theoretical expectations to isolate and quantify the various components (i.e., debris and nuclei in different stages) that contribute to the shape of the histogram. This makes it possible to account for (and remove) the contribution of debris to histogram peaks before calculating nuclei parameters. Furthermore, using nonlinear regression replaces subjective visual gating with robust numerical model‐fitting routines.

flowPloidy implements the histogram analysis procedure described by Bagwell (1993). This approach has been implemented in commercial programs such as ModFit (Verity Software House, http://www.vsh.com) and MultiCycle AV (Phoenix Flow Systems, http://www.phnxflow.com). Our implementation differs in several respects that will be useful to plant scientists: (1) flowPloidy is freely available as part of the open source R Statistical Computing Environment (R Core Team, 2017); (2) as part of the R environment, flowPloidy provides results that can be directly incorporated into further analyses within that system, without requiring users to export or convert to different formats; (3) the source code is available for inspection and modification, enabling advanced users to further customize or extend their analyses; and (4) the workflow is simplified relative to other programs, focusing only on those features necessary for genome size assessment.

## METHODS AND RESULTS

flowPloidy builds on existing R packages for processing FCM data. Specifically, it uses the read.FCS function from the flowCore package (Ellis et al., 2017) to import data. read.FCS supports the standard FCS 2.0, FCS 3.0, and List Mode Data file formats (http://www.isac-net.org). All flow cytometers we have used produce data in at least one of these formats.

After flowPloidy loads an FCM file, data from the desired channel (specified by the user) are extracted and aggregated into bins to form the histogram. A numerical model of the histogram is then constructed from a list of possible components. G1 and G2 nuclei peaks are modeled as Gaussian curves, debris is modeled using either the single‐cut or multi‐cut model, S‐phase cells are modeled as broadened rectangles, and aggregates are modeled using the continuous aggregate model. The mathematical details of each of these models are provided in Bagwell (1993), and also in the help files for flowPloidy (in particular, ?ModelComponent, ?gauss, and ?DebrisModels, accessible from the R console after flowPloidy is installed and loaded). A tutorial explanation is provided with the package (the flowPloidy vignette “histogram‐tour,” also available on the Bioconductor website [https://bioconductor.org/packages/release/bioc/vignettes/flowPloidy/inst/doc/histogram-tour.html]).

The individual model components are combined into a single model for the histogram. This combined model may contain six or more components, with a dozen or more parameters. Initial estimates for each parameter are generated by flowPloidy and used to calculate the fit. The actual model fitting is done via the nlsLM nonlinear regression function from the minpack.lm package (Elzhov et al., 2016). The residual *χ* ^2^ value is calculated as a rough goodness‐of‐fit measure (Bagwell, 1993).

**Figure 1:**
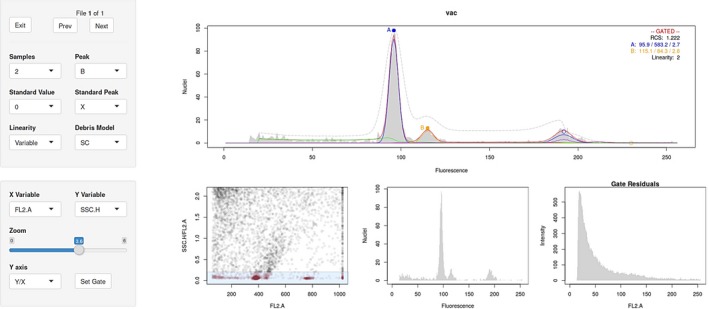
The flowPloidy histogram browser. The upper image shows the fitted histogram, whereas the lower three images display gating data, controlled by the controls at lower left. Additional analysis options and navigation tools are provided in the upper left box.

In regular use, users will want to visually inspect and correct the model fitting. flowPloidy uses the shiny package (Chang et al., 2017) to provide an interactive graphical browser for the fitted histogram models (Fig. 1). One of the advantages of the model‐fitting approach to FCM analysis is that the nlsLM algorithm is robust to small inaccuracies in initial parameter estimates. As long as the estimate is reasonably close, the result will be the same. If the estimate is not close enough, the resulting poor fit is readily apparent to the user and easily corrected by selecting more appropriate starting values, using the point‐and‐click graphical interface.

In some cases, particularly with samples with a high level of background debris, some gating is required. The flowPloidy shiny interface provides a separate window with which to zoom in on a scatterplot of the FCM data and select the region to include (gate) in the analysis. The same nonlinear regression model‐fitting process is used on the gated data. This minimizes the impact of small differences in the extent of the gate on the resulting parameter estimates. Nevertheless, gating introduces uncertainties into the model‐fitting process, which are further discussed in the flowPloidy help files (particularly ?DebrisModels).

A tutorial overview of the complete flowPloidy workflow is provided with the package (the flowPloidy vignette “flowPloidy‐gettingStarted,” also available on the Bioconductor website [https://bioconductor.org/packages/release/bioc/vignettes/flowPloidy/inst/doc/flowPloidy-gettingStarted.html]).

flowPloidy is designed with the analysis of a directory full of FCS files in mind. After a directory is loaded and evaluated, the resulting parameter estimates are stored in an R data frame object. At this point, further analysis in R, or export to a CSV file, are easily accomplished. The results of the analysis include: the total number of nuclei modeled, the size and coefficient of variation of each peak, the ratio of the sample peak to the standard peak, the residual *χ* ^2^ value, and the size of the sample genome in picograms.

flowPloidy will run on any platform that is supported by R. That currently includes 64‐bit Intel‐based Macs (i.e., Macs built since 2008), Windows 7 or later, and standard Linux desktop distributions such as Ubuntu, Debian, or Red Hat. flowPloidy is distributed through the Bioconductor repository (Huber et al., 2015), which requires the latest version of R. It can be installed from R with the following commands: 
source(“https://bioconductor.org/biocLite.R”)biocLite(“flowPloidy”)



We compared the results from flowPloidy and ModFit for 48 FCM samples selected to represent a range of genome sizes and tissue quality. This analysis combines data sets from three separate species: *Petunia **×**atkinsiana* (Sweet) D. Don ex W. H. Baxter (leaf tissue), *Malus coronaria* (L.) Mill. (mature embryos), and *Chamerion angustifolium* (L.) Holub (single whole seeds). In each case, DNA content (pg/2c) for the test species is calculated relative to an internal standard (*Solanum lycopersicum* L., *Epilobium hirsutum* L., and *Zea mays* L., respectively). Quality of the samples varied from high (*Petunia **×**atkinsiana*, CVs <3%, nuclei number/peak >1200), to moderate (*Malus coronaria*, CVs <4%, nuclei number/peak >600), to poor (*Chamerion angustifolium*, CVs = 3–9%, nuclei number/peak = 33–490). The *Chamerion* samples also had high debris counts relative to nuclei counts.

We assessed three key parameters: DNA content (i.e., the G1 peak ratio × the DNA content of the standard), the CV for the test species G1 fluorescence peak, and the number of nuclei in the G1 peak of the test species. The CV and nuclei count are recommended quality‐control measures for FCM (Suda et al., 2007a). We present here the results of Pearson correlation tests and paired *t*‐tests. The residuals were not normal for the correlations, so we have also included non‐parametric versions of these tests in Appendix S1; the results of the parametric and non‐parametric tests are very similar, and do not differ in their overall conclusions.

**Figure 2:**
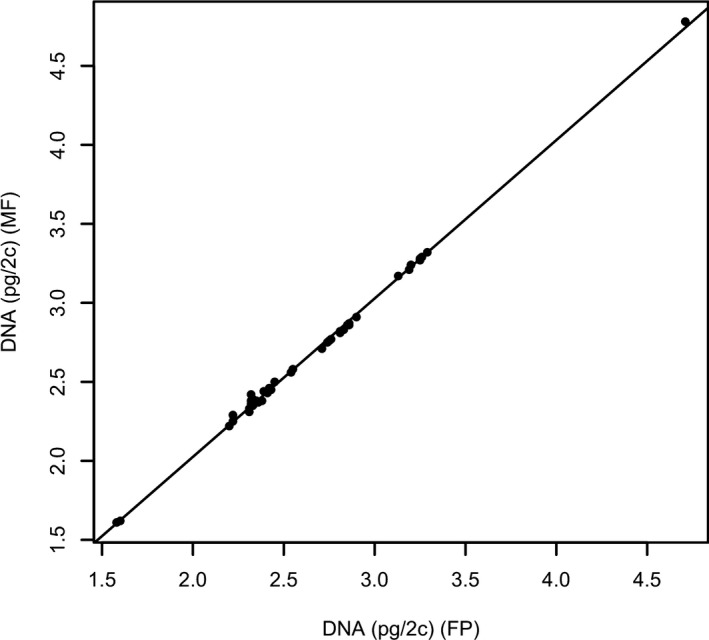
DNA content estimates for ModFit (MF,* y*‐axis) and flowPloidy (FP,* x*‐axis). Values were calculated for the same 48 samples, as described in the text. The regression line is included: MF = 0.021 + 1.002 FP,* R* ^2^ = 0.998, *P* < 0.0001.

All three parameters were highly correlated between flowPloidy and ModFit, as shown in Table 1. flowPloidy produced a slightly lower mean DNA content estimate (2.62 vs. 2.65 pg/2c; Table 2, Fig. 2), but the mean difference (0.0267 pg) is only 1% of the mean DNA content estimate. This falls well within the 3–4% error range often observed in genome size estimation with flow cytometry (Suda et al., 2007b; Bainard et al., 2011). flowPloidy produced a slightly higher mean nuclei number than ModFit. The mean difference (112) was 6.6% of the mean nuclei number. There was no statistically significant difference between flowPloidy and ModFit for the CV value (3.93 vs. 4.05; Table 2).

**Table 1:** Pearson correlation between flowPloidy and ModFit for three key flow cytometry parameters. Each correlation used the same 48 samples, as described in the text

Parameter	*R* ^2^	*P* value	Slope	Intercept
DNA content	0.998	<0.0001	1.002	0.021
CV	0.957	<0.0001	1.093	−0.251
Nuclei	0.993	<0.0001	0.911	43.83

Note

CV = coefficient of variation.

**Table 2:** Paired *t*‐tests comparing flowPloidy and ModFit for three key flow cytometry parameters. Each test used the same 48 samples (*df* = 47), as described in the text

Parameter	flowPloidy mean	ModFit mean	*t*	*P* value
DNA content	2.62 pg	2.65 pg	−8.85	<0.0001
CV	3.93%	4.05%	−1.56	0.1253
Nuclei	1747	1635	4.05	0.0002

Note

CV = coefficient of variation.

We note that it is not unexpected to find small differences between the flowPloidy and ModFit analyses. The model‐fitting procedure described by Bagwell (1993) is complex, and the relationships between all parameters are not completely defined. Consequently, our implementation will differ in minor ways. Despite this, the differences documented here, while statistically significant, are not biologically meaningful. Indeed, we routinely observe differences of similar or greater magnitude between replicate runs of the same samples.

We now use flowPloidy in preference to other FCM software in our lab. flowPloidy is more convenient to use, as it can be installed on multiple computers at no cost; it is a fully integrated component of our R‐based analyses. The simpler interface is also easier to use and requires less training time for new users.

## CONCLUSIONS

Nonlinear‐regression‐based FCM histogram analysis is the most objective and repeatable way to determine genome size for plants. flowPloidy provides convenient access to this powerful analytical approach. By making this technique widely available, we hope to encourage plant scientists to adopt it as part of FCM best practice, ensuring reliable and repeatable analyses.

## DATA ACCESSIBILITY

flowPloidy is available on the Bioconductor repository (https://bioconductor.org), with links to the source code, development repository, and documentation at the flowPloidy landing page (https://bioconductor.org/packages/release/bioc/html/flowPloidy.html; https://doi.org/10.18129/b9.bioc.flowploidy).

## Supporting information

Appendix S1
