# Supplementary material for: flowPloidy: An R package for genome size and ploidy assessment of flow cytometry data
Source: Appl Plant Sci. 2018 Jul 23;6(7):e01164. doi: 10.1002/aps3.1164 (PMC6055564; doi:10.1002/aps3.1164)

APPENDIX S1. A comparison of ploidy analysis results generated by the programs flowPloidy and ModFit.

This analysis uses a data set that combines data sets from three separate species: *Petunia ×atkinsiana* (leaf tissue), *Malus coronaria* (mature embryos), and *Chamerion angustifolium* (single whole seeds). In each case, DNA content (pg/2c) for the test species is calculated relative to an internal standard (*Solanum lycopersicum*, 1.96 pg/2c; *Epilobium hirsutum*, 0.84 pg/2c; and *Zea mays*, 5.43 pg/2c, respectively). Quality of the samples varied from high (*P. ×atkinsiana*, CVs <3%, nuclei number/peak >1200), to moderate (*M. coronaria*, CVs <4%, nuclei number/peak >600), to poor (*C. angustifolium*, CV = 3–9%, nuclei number/peak = 33–490). The *C. angustifolium* samples also had high debris counts relative to nuclei counts.

DNA CONTENT

Summary

DNA content estimates as estimated with flowPloidy and ModFit were highly correlated, with an $R^2 = 0.972$ ($P < 0.0001$; Spearman's) and the best fit line having a slope of 1.002. flowPloidy produced a slightly lower mean DNA content estimate (2.62 vs. 2.65 pg/2c, $P < 0.001$), but the mean difference (0.0267 pg) is only 1% of the mean DNA content estimate. This falls well within the 3–4% error range often observed in genome size estimation with flow cytometry.

Test for correlation:

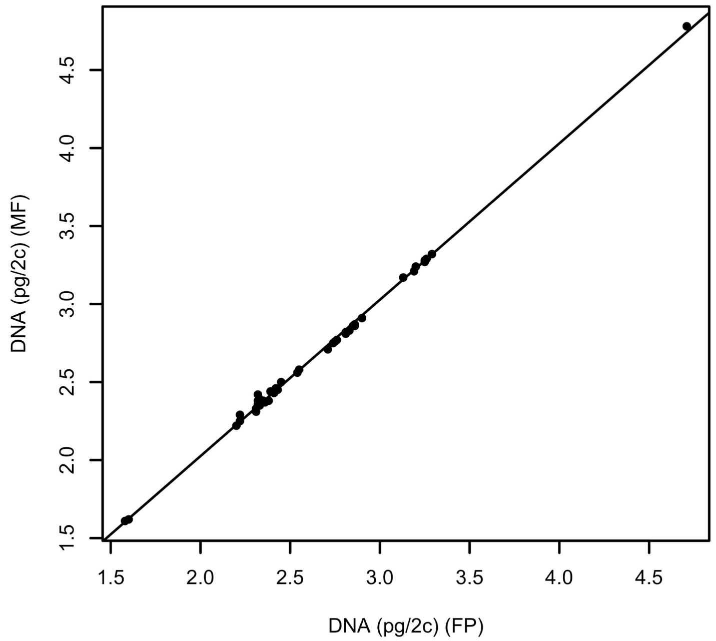

Best fit line: $y = 0.020646 + 1.002296\,x$.
Pearson's: $R = 0.999139$, $R^2 = 0.998278$
$t = 163.29$, $df = 46$, P value < 2.2e-16

DOI

N = 48 Confidence interval (0.9985, 0.9995).
Residuals not normal, P = 9e-04
Spearman's: rho = 0.9859266
S = 259.29, P value < 2.2e-16

Test for equality of means:

Mean DNA content (pg/2c)

FP	MF
2.62	2.65

Paired *t*-test
t = −8.8535, df = 47, ***P* value = 1.4e-11 (<0.001)**
Mean of difference (FP-MF) and 95% CI: −0.0267 (−0.0327, −0.0206)

Non-parametric *t*-test
Wilcoxon signed rank test with continuity correction
V = 0, ***P* value = 1.523e-08**

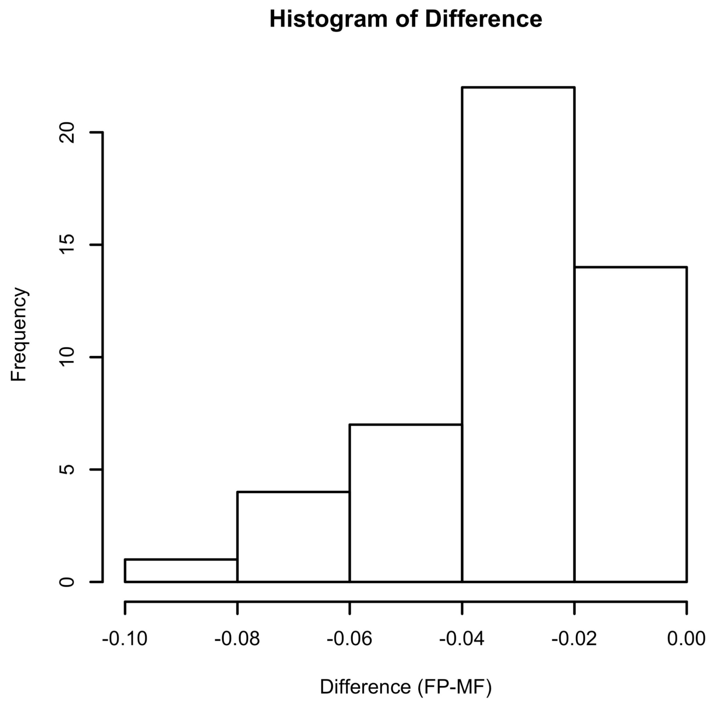

COEFFICIENTS OF VARIATION (OF THE TEST SPECIES G1 PEAK)

Summary

The CVs for the G1 peak of the test species as measured with flowPloidy and ModFit were highly correlated, with an $R^2 = 0.9665$ ($P < 0.0001$) and the best fit line having a slope of 1.093. flowPloidy produced a slightly lower mean CV than ModFit, but the difference was not statistically significant ($P = 0.125$, $P = 0.4542$ for non-parametric).

Test for correlation:

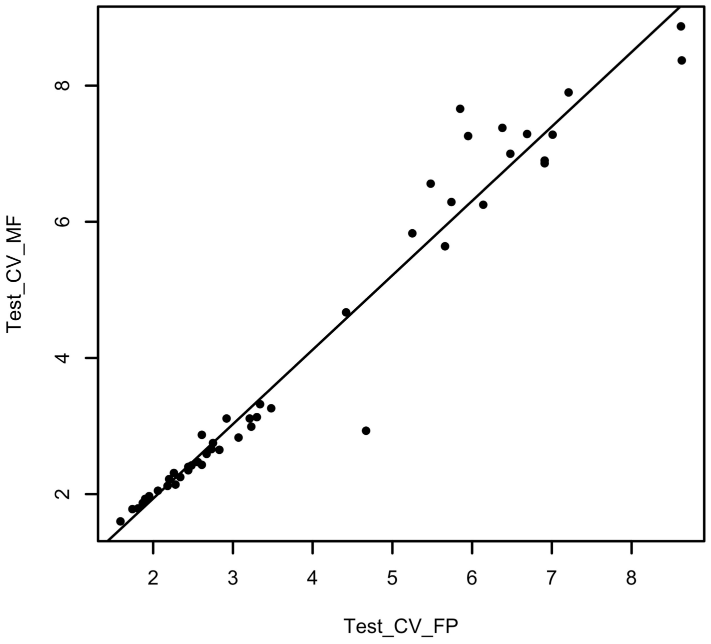

Best fit line: $y = -0.251631 + 1.092971\,x$.
Pearson's: $R = 0.978641$, $R^2 = 0.957739$
$t = 32.287$, $df = 46$, ***P* value < 2.2e-16**
$N = 48$ Confidence interval (0.962, 0.988).
Residuals not normal, $P = 0$
Spearman's rank correlation rho
S = 311.54, *P* value < 2.2e-16
Rho = 0.9830904

Test for equality of means:

Mean CV (%)

FP	MF
3.93	4.05

Paired *t*-test
$t = -1.5605$, $df = 47$, P value = 0.1253
Mean of difference (FP-MF) and 95% CI: −0.12 (−0.261, 0.0330)

Non-parametric paired test
Wilcoxon signed rank test with continuity correction
V = 471.5, P value = 0.4542

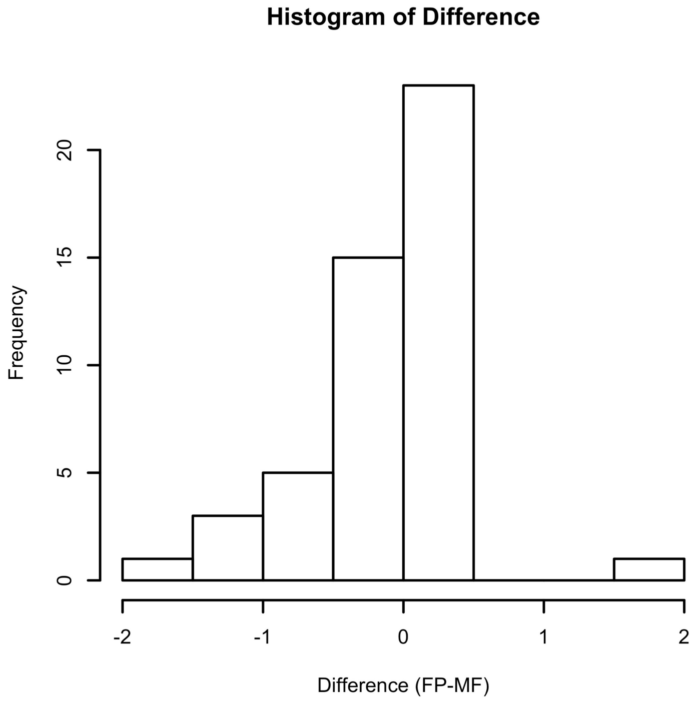

NUCLEI NUMBER IN G1 PEAK (OF THE TEST SPECIES)

Summary

The number of nuclei in the G1 peak of the test species as measured with flowPloidy and ModFit were highly correlated, with rho = 0.9933782 ($P < 0.0001$) and the best fit line having a slope of 0.91. flowPloidy produced a slightly higher mean nuclei number than ModFit ($P < 0.0001$). The mean difference (112) was 6.6% of the mean nuclei number (1747 for flowPloidy, 1635 for ModFit).

Test for correlation:

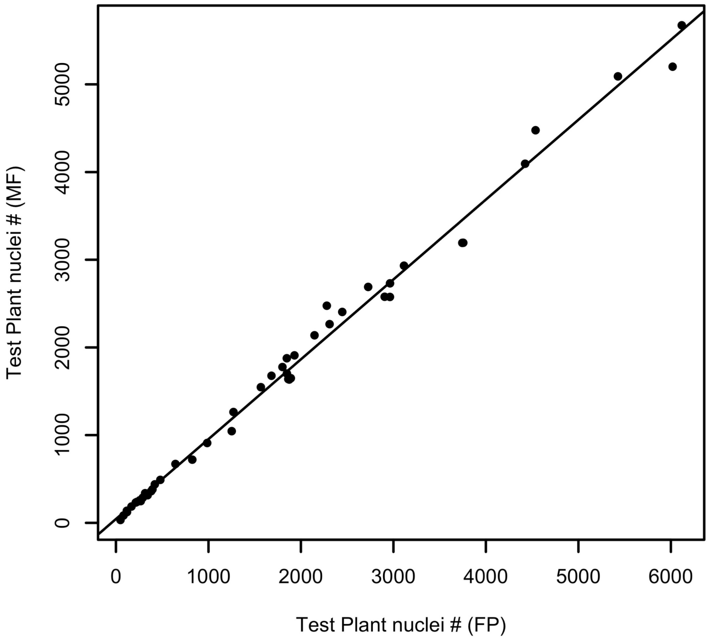

Best fit line: $y = 43.825749 + 0.910529\, x$.
Pearson's: $R = 0.996458$ $R^2 = 0.992929$
$t = 80.369$, $df = 46$, ***P* value < 2.2e-16**
$N = 48$ Confidence interval (0.994, 0.998).
Residuals not normal, $P = 0.0476$
Spearman's rank correlation rho
S = 122, *P* value < 2.2e-16
rho 0.9933782

Test for equality of means:

Mean CV (%)

FP	MF
1747	1635

Paired *t*-test
$t = 4.0454$, $df = 47$, ***P* value = 0.0001931**
Mean of difference (FP-MF) and 95% CI: 112.5 (56.56, 168.46)

Non-parametric paired test
Wilcoxon signed rank test with continuity correction
V = 973, *P* value = 3.571e-05

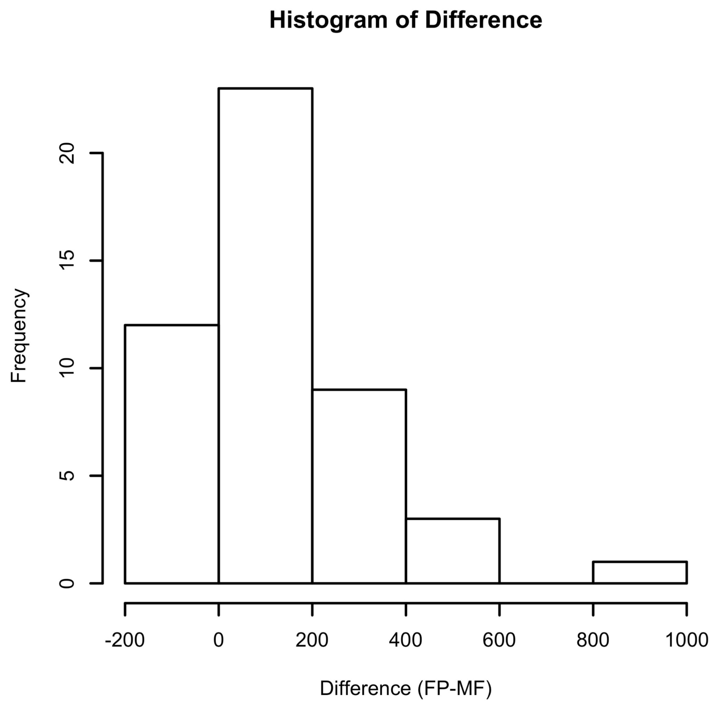

Supplement: Supplementary file 1 — Appendix S1 [file APS3-6-e01164-s001.pdf]
